# PD-L1 predicts poor prognosis for nasopharyngeal carcinoma irrespective of PD-1 and EBV-DNA load

**DOI:** 10.1038/srep43627

**Published:** 2017-03-03

**Authors:** Yajuan Zhou, Dingbo Shi, Jingjing Miao, Haijun Wu, Jiewei Chen, Xiaoyi Zhou, Desheng Hu, Chong Zhao, Wuguo Deng, Conghua Xie

**Affiliations:** 1 Hubei Key Laboratory of Tumour Biological Behaviors, Department of Radiation and Medical Oncology, Zhongnan Hospital of Wuhan University, Wuhan, China; 2 Department of Radiation Oncology, Hubei Cancer Hospital, Wuhan, China; 3 Collaborative Innovation Center for Cancer Medical, State Key Laboratory of Oncology in South China, Sun Yat-sen University Cancer Center, Guangzhou, China; 4 Department of Nasopharynx, Collaborative Innovation Center for Cancer Medical, State Key Laboratory of Oncology in South China, Sun Yat-sen University Cancer Center, Guangzhou, China; 5 Department of Pathology, Collaborative Innovation Center for Cancer Medical, State Key Laboratory of Oncology in South China, Sun Yat-sen University Cancer Center, Guangzhou, China

## Abstract

Programmed death-1 (PD-1) is an immunosuppressive receptor functionally bound with programmed death-ligand 1 (PD-L1), which has been reported in various malignancies. However, only a few studies are available for the clinical significance of PD-1/PD-L1 in nasopharyngeal carcinoma (NPC). In this study, we aim to investigate alterations in PD-1/PD-L1 by using immunohistochemistry analysis in a cohort of consecutively enrolled NPC patients (n = 99). To further analyse the correlation between PD-1/PD-L1 and factors involved in clinico-pathology, haematologic biomarkers, EBV-DNA load and outcomes, we collected clinical data for statistical analysis. We observed that lower haemoglobin (HB) and Body Mass Index (BMI) levels were associated with high levels of PD-L1 staining in NPC patients. Importantly, our results suggested that PD-L1 might be a negative indicator for NPC patients. In contrast, a correlation between the PD-1/PD-L1 level and EBV load was not identified. Moreover, PD-1 positivity was suggested to not be significantly correlated with clinical outcomes. Taken together, our results revealed that PD-L1 might be a potential prognostic biomarker for NPC patients. However, further studies are needed to clarify the underlying mechanism of EBV status in the immunosuppression process induced by the PD-1/PD-L1 axis.

Nasopharyngeal carcinoma (NPC) is an Epstein-Barr virus (EBV)-related malignancy, with an annual incidence of 30–80 per 10,000 in endemic regions, such as South China^1^. NPC is highly sensitive to radiotherapy and chemotherapy. Despite the development of precise radiotherapy technologies and combined chemotherapy, treatment failure for NPC remains quite frequent, with rates of approximately 10% for recurrence and 20% for distant metastasis^2^,^3^. Better and novel treatments are urgently needed to improve survival.

NPC is characterized by substantial lymphocytic infiltration (mainly of T cells) in the primary tumour^4^,^5^. Programmed death-1 (PD-1 or CD279) is an immunosuppressive receptor that is expressed in T cells^6^. PD-1 engagement by programmed death-ligand 1 (PD-L1 or CD274) in cancer cells decreases T cell activation and induce tumour immune escape^6^,^7^. It has been generally recognized that PD-1/PD-L1 protein abundance is associated with aggressive histology and worse prognosis in multiple tumour types, such as PD-1 in breast cancer^8^ and soft tissue sarcomas^9^, as well as PD-L1 in melanoma^10^ and renal cell carcinoma^11^. However, contradictory prognostic values have also been reported in several tumours. For example, PD-1-stained T cells were correlated with improved survival in follicular lymphoma^12^ and HPV-associated head and neck cancer^13^, and PD-L1 expression was associated with a better prognosis of pulmonary squamous cell carcinomas^14^ and breast cancer^15^. However, until recently, only a few studies on the PD-1/PD-L1 axis in NPC are available, and the reported prognostic role of PD-L1 in NPC patients remains inconsistent. For instance, Zhang *et al*.^16^ revealed that PD-L1 protein abundance was correlated with a worse outcome. In contrast, a positive correlation of PD-L1 expression and survival of non-metastatic NPC was delineated by Lee *et al*.^17^. In addition, Hsu *et al*.^18^ showed that PD-1 on CD8 T cells predicted a poor prognosis in a small size of NPC patients, whereas PD-1 expression might not be a prognostic factor in NPC patients when using a larger sample^16^. Therefore, the prognostic role of PD-1 and PD-L1 in NPC needs to be clarified.

The blockade of the PD-1 and PD-L1 pathway is one of the most promising strategies to activate anti-tumour immunity^19^. There are also ongoing clinical trials evaluating the safety and efficiency of anti-PD1 antibodies in NPC^20^. Clinical data have shown that the treatment response to PD-1/PD-L1 blockade was correlated with PD-1 or PD-L1 detected by immunohistochemistry (IHC)^21^,^22^. In addition to PD-1/PD-L1 expression, many host-related influences might account for the heterogeneous responses and failures during immunotherapies, such as nutritional status^23^, smoking^24^, and inflammation status^25^. The tumour microenvironment, including hypoxia and immunosuppressive cytokine production, also plays a role in the therapeutic response^26^. Baseline peripheral blood parameters have been reported as predictive biomarkers for the response to PD-1 antibodies^27^. Thus, additional information about the clinico-pathological characteristics and haematologic biomarkers related to PD-1/PD-L1 expression could promote a better understanding of their regulatory role in NPC and might be helpful for improving the response to anti PD-1/PD-L1 treatment.

In addition, PD-1 and PD-L1 could induce T cell deactivation during chronic viral infections^28^,^29^. The Epstein-Barr virus (EBV) has been reported to be strongly linked with NPC in epidemic areas^30^. EBV-DNA load significantly correlates not only with NPC development but also with treatment failure^31^. Experimental studies^32^ have suggested that there is an upregulation of PD-L1 in EBV-positive NPC cell lines. Despite improvement in preclinical research, only a little information is currently available for the correlation between EBV-DNA load and PD-1/PD-L1 expression in NPC.

Taken together, the prognostic value of PD-1/PD-L1 expression in NPC remains largely unknown. Detailed data on potential related biomarkers and EBV infection status with PD-1/PD-L1 are needed to be confirmed by clinical data. Therefore, considering the tremendous therapeutic potential of targeting the PD-1/PD-L1 interaction in NPC, we analysed the prognostic value of PD-1 and PD-L1 in a cohort of NPC patients (n = 99), which were consecutively recruited form a single medical care group over the course of two years. We also evaluated the correlation between PD-1/PD-L1 expression and clinico-pathological parameters as well as potential related haematologic biomarkers, such as the haemoglobin (HB) level. Our study might provide a new strategy for immune checkpoint-blocking therapy.

## Results

### General information

All of the nasopharynx biopsy samples (n = 99) were collected and histologically diagnosed as NPC by experienced pathologists. The samples were fixed with formalin and embedded into paraffin using a tissue processor. Among the 99 patients enrolled, including 68 males and 31 females, the median age was 47.5 years old (range from 20 to 78 years). All patients were treated with intensity-modulated radiation therapy (IMRT) with or without combined chemotherapy. The average follow-up time was 49.4 months (range from 6.7 to 64.9 months). In this cohort, 18 patients encountered disease progression (recurrence and/or metastasis), and 9 patients died during the follow-up period. All tumours were classified as the undifferentiated non-keratinizing phenotype. The characteristics of the enrolled patients are summarized in Supplementary Table S1.

### Clinico-pathologic correlations

**Figure 1:**
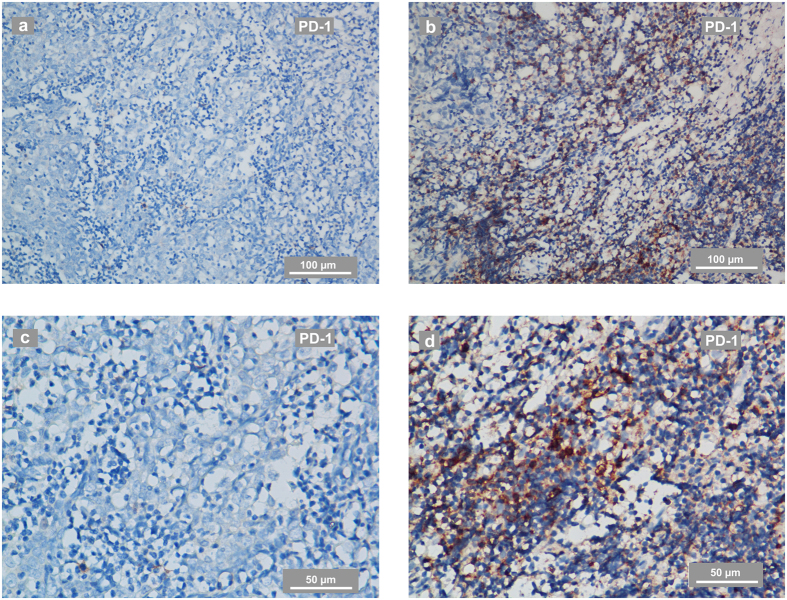
Representative IHC staining of PD-1 in NPC-biopsies. (**a**,**c**) PD-1 staining in a biopsy from an NPC patient evaluated as PD-1 negative. The IHC photos were taken using phase-contrast microscopy and are shown at low (**a**) and high magnification (**c**). (**b**,**d**) PD-1 staining in a biopsy from an NPC patient evaluated as PD-1-positive. The IHC photos are shown at low (**b**) and high magnification (**d**). The scale bars for a-b are 100 μm and for c-d are 50 μm.

**Figure 2:**
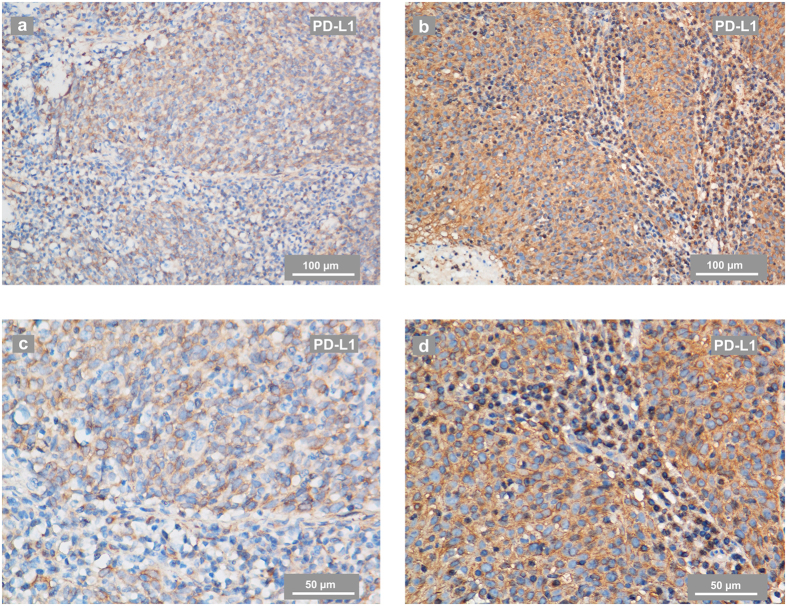
Representative IHC staining of PD-L1 in NPC-biopsies. (**a**,**c**) PD-L1 staining in a biopsy from an NPC patient evaluated as having a low level of PD-L1. The IHC photos were taken using phase-contrast microscopy and are shown at low (**a**) and high magnification (**c**). (**b**,**d**) PD-L1 staining in a biopsy from an NPC patient evaluated as having a high level of PD-L1. The IHC photos are shown at low (**b**) and high magnification (**d**). PD-L1 expressed in tumour-infiltrating lymphocytes in a scattered manner. The scale bars for a-b are 100 μm and for c-d are 50 μm.

PD-1-positive immune cells were present in 44 of the total 99 tumours (44.4%), and PD-L1 staining was detectable in 96 patients (97.0%) and was mainly located at the membrane or in the cytoplasm region (or both) in the tumour cells. PD-L1 was also expressed in tumour-infiltrating lymphocytes (TILs) in a scattered manner. According to the ROC curve analysis for OS, the optimal cut-off value for the H-score was 155 (AUC: 0.780, sensitivity: 1.000, specificity: 0.419). Thus, when the H-score ≥155, PD-L1 staining was classified as being at a high level (61 cases). PD-1 positivity was defined as cases with PD-1 staining intensity ≥2 in more than 5% of TILs. Representative stainings of PD-1 and PD-L1 in NPC are shown in Figs 1 and 2. The optimal cut-off value for the nasopharynx gross tumour volume (GTVnx) was 29.6 mm^3^ (AUC: 0.740, sensitivity: 0.917, specificity: 0.505). In this study, neither PD-1 positivity nor a high level of PD-L1 staining was significantly correlated with the clinico-pathological parameters of age, gender, smoking status, family history of cancer, GTVnx or clinical stage at diagnose. Detailed data are summarized in Table 1

### BMI and HB in 99 NPC patients with heterogeneous immunoreactivity of PD-1/PD-L1

**Figure 3:**
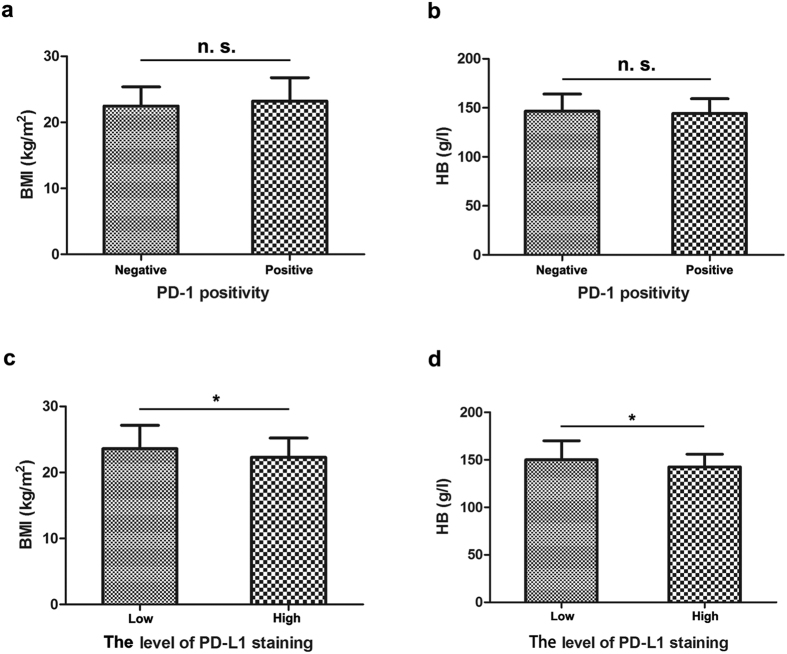
BMI and HB in 99 NPC patients with different PD-1/PD-L1 levels. (**a,b**) Statistical analysis suggested no significant (n. s.) differences in the BMI (**a**) or HB (**b**) levels between patients with positive or negative PD-1. (**c,d**) Statistical analysis revealed a significantly lower BMI (**c**) and HB (**d**) level in patients with high levels of PD-L1. A t-test was used to evaluate the association of PD1/PD-L1 with BMI and HB level after normality tests and homogeneity of variance test. *P < 0.05.

Weight and height were collected to calculate the body mass index (BMI), calculated by dividing the weight in kilograms by the square of the height in metres (kg/m^2^). The BMI of the 99 NPC patients ranged from 16.26 to 33.83 kg/m^2^. We compared the difference in the BMI level of patients with low or high levels of PD-L1 staining, and our results suggested that patients with a high level of PD-L1 staining had significantly lower BMI levels (P = 0.048, Fig. 3c). Our results also revealed that the HB level was significantly reduced in the patients with a high level of PD-L1 staining (P = 0.029, Fig. 3d). In contrast, we observed no significant difference in the BMI (a) or HB level (b) in patients with positive or negative PD-1 staining. (Fig. 3a,b).

### No significant association between PD-1/PD-L1 expression and EBV load

**Figure 4:**
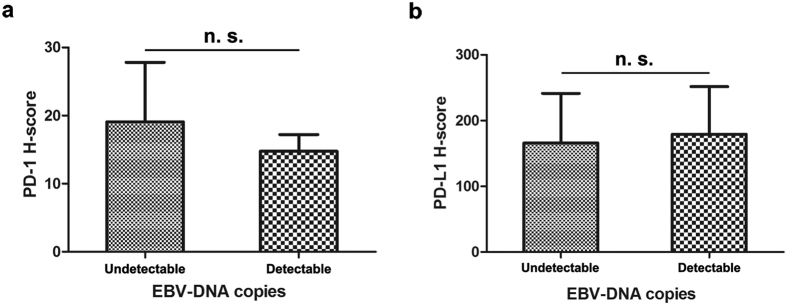
No significant differences in the PD-1 or PD-L1 H-score between NPC patients with undetectable or detectable baseline EBV-DNA copies Statistical analysis shows no significant (n. s.) difference in the PD-1 H-score (**a**) or the PD-L1 H-score (**b**) between patients with undetectable or detectable EBV loads. The differences were evaluated by non-parametric Mann-Whitney U test.

To evaluate the correlation between EBV viral load and immunoreactivity of PD-1 and PD-L1 in the NPC patients, we collected the qRT-PCR data of EBV-DNA copies from our database. In this cohort of 99 patients, EBV-DNA load was detectable in 77 patients. Non-parametric tests showed no significant difference in the PD-1 (P = 0.853, Fig. 4a) or PD-L1 H-scores (P = 0.389, Fig. 4b) between patients with a detectable or undetectable EBV-DNA load. Chi-square tests indicated that there was no significant association between the PD-1 (P = 0.914, Table 1) or PD-L1 level (P = 0.439, Table 1) and the EBV level.

### Prognostic values related with PD-1 and PD-L1

**Figure 5:**
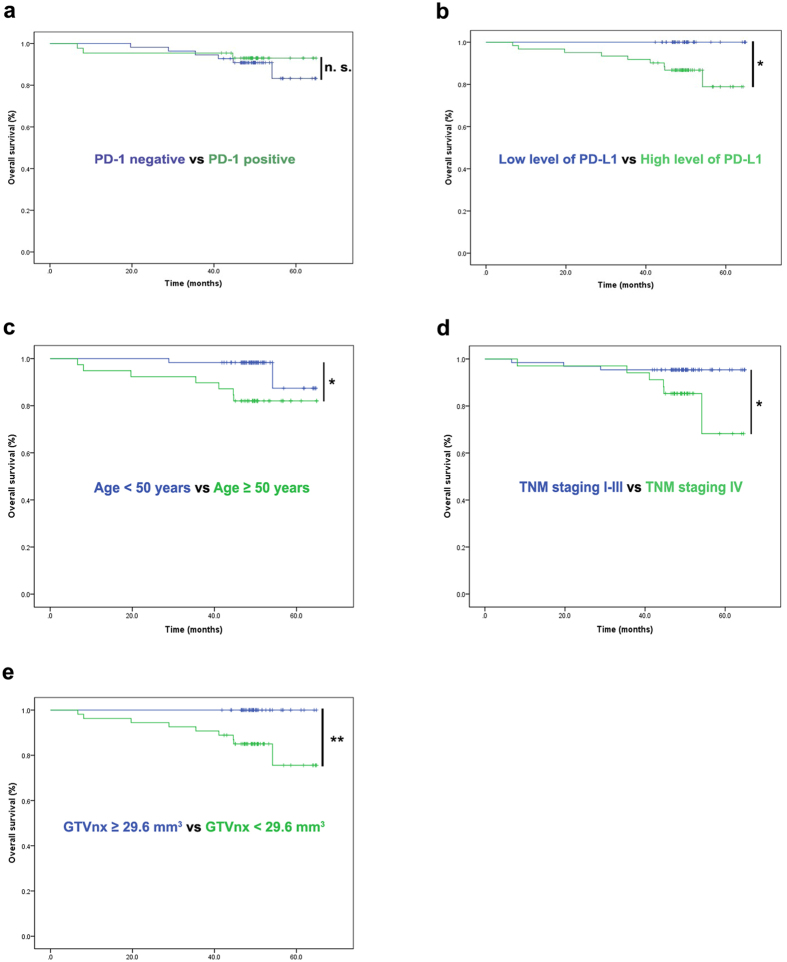
Prognostic factors of the 99 NPC patients by Kaplan-Meier survival analysis. (**a**) Statistical analysis suggested that PD-1 positivity on TILs was not significantly correlated with overall survival (OS). The results revealed significant associations between OS and high levels of PD-L1 (**b**), age (**c**), TNM staging (**d**) and GTVnx (**e**). The cut-off points for PD-L1 and GTVnx were generated from the ROC. Survival analysis used the Kaplan-Meier method. Univariate analysis was performed with a log-rank test. *P < 0.05, **P < 0.01.

To evaluate the prognostic values in PD-1-positive tumour-infiltrating lymphocytes (TILs) and tumour cells with PD-L1 expression, we used the Kaplan-Meier survival analysis and log-rank tests. In this consecutively enrolled cohort of 99 NPC patients, our results revealed that a high expression of PD-L1 was correlated with shorter OS (P = 0.015, Fig. 5b) and showed trend of a reduced progression-free survival rate (PFS rate) (P = 0.127, Table 2). The factors significantly correlated with OS by univariate analyses were age (Fig. 5c), the level of PD-L1 staining (Fig. 5b), clinical stage (Fig. 5d), and GTVnx (Fig. 5e) before treatment. In contrast, we observed that the PD-1 positivity in TILs was not significantly correlated with OS (P = 0.563, Fig. 5a) or PFS (P = 0.616, Table 2). Moreover, in the PD-L1 highly expressed cases, the positivity of PD-1 could not further predict the OS (P = 0.399) and PFS of patients (P = 0.956). Multivariate analyses of prognostic factors were performed using a Cox regression model. T classification was not put in the multivariate analysis for it was found to be significantly correlated with clinical stage by a chi-square test (φ correlation coefficient = 0.868, P = 0.000) in this group of NPC patients. Thus, the variables included in the multivariate analysis for OS were age, clinical stage, GTVnx and PD-L1 H-score (Table 3). In the multivariate analysis, only the PD-L1 H-score was suggested to be an independent prognostic factor for OS. Patients with a higher PD-L1 H-score could have a greater risk of death (95% CI, 1.002–1.031, P = 0.028, Table 3).

## Discussion

The association of the immunological checkpoint PD-1/PD-L1 and its prognosis of various cancers are currently a research hotspot^19^. PD-L1 has been reported to be overexpressed in most tumours including NPC^16^,^33^ to inhibit T cell-mediated antitumour immunity via PD-1 on TILs^34^. Recent studies have also revealed that PD-L1 was associated with poor prognosis in most epithelial-originated cancers^35^, suggesting an effect of PD-L1 in the induction of tumour progression by interrupting anti-tumour immunity^36^. However, the prognostic significance of PD-L1 as well as PD-1 in NPC has not been clarified yet. We collected biopsies and clinical data from 99 consecutively enrolled NPC patients to analyse the association of PD-L1/PD-1 and related factors. Our results suggested that NPC patients with a high level of PD-L1 had a significantly reduced survival outcome (Fig. 5b), which is consist with the results from 132 NPC patients^16^. However, another publication noted that there was a longer survival rate in non-metastatic NPC patients with a high PD-L1 level^15^, which might be explained by heterogeneous applications of different experimental procedures, scoring criteria, patient samples and survival endpoints. These differences could also illustrate why the PD-L1 expression rate was found to be much lower (25%) in the NPC patients reported by Lee *et al*.^17^ than that reported in our results (97.0%) and Zhang *et al*.^16^ (95.0%).

Furthermore, we also analysed the correlation between PD-1 positivity and the survival outcomes of NPC patients and found that there was no significant association (Fig. 5). Interestingly, previous publications have suggested a worse clinical outcome in NPC patients with PD-1-positive CD8 T cells (total n = 46)^18^. However, in a larger cohort (total n = 132), the impact of PD-1 was not obvious^16^.

As of now, standardized testing procedures and consistent scoring criteria for PD-1/PD-L1 in solid malignancies by IHC still need to be optimized^35^,^37^,^38^,^39^. The H-score has been applied in many IHC studies for its semi-quantitative characteristic^39^,^40^. Since no standard scoring criteria for PD-1 and PD-L1 are available^35^,^38^,^41^, we have used an ROC analysis to determine the cut-off value for PD-1 and PD-L1. However, our results suggested no significant cut-off value for PD-1 based on survival endpoints. The cut-off point was 155 for the PD-L1 H-score by ROC analysis in this study, while an intensity ≥2^17^ or an H score ≥35^16^ was chosen to be the cut-off value in previous publications. As reported by Zhang *et al*.^16^, an H-score > 0 was applied as the cut-off value for PD-1. Consistently, we also found no significant correlation with the pathological features or prognostic role of PD-1 when the cut-off was defined as an H-score > 0. We raised the criteria of PD-1 positivity to a staining intensity ≥ 2 in more than 5% of the TILs (H-score ≥ 10), similar to the methods reported by D’Incecco *et al*.^24^, to minimize the potential interference of non-specific staining for PD-1.

Our study revealed a non-significant difference between the PD-L1 level and PD-1 positivity (Table 1), suggesting an indirect interaction of PD-L1 and PD-1 in NPC cells. PD-1 belongs to the CD28 family and is characterized as an inhibitory receptor expressed in T cells, dendritic cells (DC), natural killer (NK) cells, macrophages and B cells^42^. The PD-1 ligands, PD-L1 and PD-L2, belong to the B7 superfamily^43^. However, binding targets of PD-L1 and PD-L2 are not restricted to PD-1, since PD-L1 and PD-L2 can also bind to CD80^44^ and Repulsive Guidance Molecule b (RGMb)^45^, respectively. In addition, PD-L1 may be expressed in T cells, B cells, myeloid dendritic cells (DC) and in tissue macrophages in the tumour microenvironment^42^. It has also been reported that the predictive significance of PD-L1 can vary depending on if the positivity is defined on tumour cells or TILs^46^,^47^. We have found an increased level of PD-L1 in TILs (Fig. 2), and further studies are needed to clarify the underlying mechanism.

The identification of potential factors that can stratify a response to anti-PD-1 therapies will aid in the selection of NPC therapy. In NPC, experimental research has demonstrated an upregulation of PD-L1 by EBV-induced latent membrane protein 1 (LMP1) in EBV-positive NPC cell lines^32^. Recently, PD-L1 and PD-1 expression were found to be significantly associated with EBV-associated malignancies^47^,^48^,^49^, where EBV-encoded RNA was used to test for EBV infection in most studies. However, our results revealed no significant correlations between baseline EBV load and PD-1/PD-L1 expression (Table 1 and Fig. 4). One possible explanation could be that the circulating cell-free plasma EBV-DNA load might be an inactive remnant of a previously active EBV infection^50^. Moreover, our study also showed reduced BMI and HB levels in patients with high levels of PD-L1 staining (Fig. 3). Because of a large overlap in confidence intervals of our statistical results, the clinical significance between these factors still needs to be confirmed in larger samples. Notably, previous studies have confirmed that a low baseline HB is an adverse prognostic factor in patients with locally advanced head and neck squamous cell carcinoma^51^ and NPC^52^, by inducing treatment resistance via enhancing tumour hypoxia. Thus, we showed these findings to arouse attention for the possible biologic relationship between HB and PD-L1, which might be triggered by hypoxia, inducing immune deficiency through the PD-1/PD-L1 axis^53^. In addition, we have also compared PD-1/PD-L1 expression with a few baseline haematological parameters, such as neutrophil counts and the lactate dehydrogenase level from peripheral blood in this exploratory analysis, and there was no significant association observed for any of the relevant factors (Supplementary Fig. S1 and S2).

Our study revealed the prognostic role of age, PD-L1, GTVnx, T classification and clinical stage in the 99 NPC patients by log-rank test. Age, smoking history, EBV-DNA load, HB, GTVnx, and T and N classification as well as clinical stage have been reported to have a prognostic role in primary NPC patients in previous studies^52^,^54^,^55^,^56^,^57^. In our study, age 50 was chosen to be the cut-off value according to previous studies on NPC patients as reported by Chen *et al*.^54^. The baseline EBV-DNA loads were dichotomized to undetectable and detectable levels as reported by Liu *et al*.^56^. The optimal cut-off point of GTVnx (29.6 mm^3^) was determined by an ROC analysis in this study, which was consistent with previous studies in different NPC samples, where 19 mm^3^ (Guo *et al*.^57^) and 25 mm^3^ (Wu *et al*.^58^) were determined to be the optimal cut-off value by ROC analysis. Patients with T1-3 classification showed a similar survival rate in this study, which might be due to the satisfactory local control rates of NPC patients raised by IMRT^59^ and might be the reason that our multivariate analysis failed to identify the clinical stage as a significant prognostic factor.

In conclusion, PD-L1 and PD-1 are widely expressed in NPC tissue. However, PD-1 positivity could not predict prognosis in our study. Importantly, PD-L1 has been suggested to be a negative prognostic factor for NPC patients. Further studies with a larger sample size are needed to confirm these observations and to evaluate the predictive value of PD-1 and PD-L1 in NPC in the context of PD-1 inhibition as well. Moreover, more studies are warranted to identify the molecular mechanism of the interaction between HB, EBV and PD-1/PD-L1 in NPC.

## Materials and Methods

### Ethics Statement

The study was approved by the Institutional Review Board of Sun Yat-sen University Cancer Center. As this was a retrospective analysis of routine data, we requested and were granted a waiver of individual informed consent from the ethics committee. Patient records/information was anonymized and de-identified prior to analysis.

### Patients and samples

For this study, 115 NPC patients were consecutively sampled by one medical care group at Sun Yat-sen University Cancer Center (Guangzhou, China) from December 2010 to December 2012. The cases were selected based on the following criteria: (1) histologically proven NPC with available biopsy specimens; (2) complete clinical data; (3) a new diagnosis; (4) non-distant metastatic NPC; (5) no treatment history or history of other malignant disease; (6) Karnofsky score ≥ 70; (7) received IMRT at Sun Yat-sen University; (8) regular follow-up. Patients with active hepatitis, diabetes, or who failed the follow-up requirements were excluded. Therefore, there were 99 cases that qualified for this study. All medical records were reviewed retrospectively, and all patients were restaged according to the 7^th^ edition of the UICC/AJCC system. FFPE blocks of NPC were retrieved by fibrescope biopsy from the Department of Pathology at Sun Yat-sen University Cancer Center. The related clinical data of these patients were retrospectively collected from our database or hospital records, including gender, age, body weight, height, clinical stage, GTVnx (gross tumour volume of primary tumour), pre-therapy laboratory counts of neutrophils, lymphocytes, HB, platelets, albumin, lactate dehydrogenase (LDH), and EBV-DNA copies by quantitative PCR test, as well as follow-up records. All of the patients were followed-up for at least 40 months.

### Radiotherapy

All patients received a computed tomography simulation scan at the radiotherapy position. The images included plain and enhanced computed tomography scans. The scope of each scan was from the top of the head to 2 cm below sub-clavicle head (thickness: 3 mm per slice). According to the institutional treatment protocol from Sun Yat-sen University Cancer Center, contouring of targets and organs at risk was performed under the IMRT planning system. The prescribed doses for the NPC patients were applied as follows: (1) planning target volume (PTV) of the primary gross tumour volume (GTVnx): 66–72 Gy at 2.12–2.43 Gy/fraction; (2) PTV of the GTV of the involved lymph nodes (GTVnd): 64–70 Gy; (3) PTV of the high-risk clinical target volume (CTV1): 60–63 Gy; (4) PTV of the low-risk clinical target volume (CTV2): 54–56 Gy. All targets were treated simultaneously using a simultaneous integrated boost technique.

### Chemotherapy (CT)

According to the institutional treatment protocol from Sun Yat-sen University Cancer Centre, IMRT was applied for stage I disease as a sole treatment, concurrent chemoradiotherapy for stage II disease, and concurrent chemoradiotherapy (CCRT) +/− neoadjuvant/adjuvant chemotherapy for stage III to IVA-B disease. Among the 99 patients involved in this study, 59 patients (59.6%) received both neoadjuvant chemotherapy (NCT) and concurrent chemotherapy (CCRT); 2 patients (2.0%) received sole NCT; 28 patients (28.3%) received CCRT; and 10 (10.1%) patients received IMRT as a sole treatment. Neoadjuvant chemotherapy consisted of cisplatin (100 mg/m^2^) with 5-fluorouracil (4.0 g/m^2^), cisplatin (75 mg/m^2^) with toxoids (75 mg/m^2^) or triple agent treatment with cisplatin (70 mg/m^2^), 5-fluorouracil (3.0 g/m^2^) and toxoids (70 mg/m^2^) every three weeks for two or three cycles. Concurrent chemotherapy consisted of cisplatin (100 mg/m^2^) given in week 1, 4, and 7 of radiotherapy.

### Immunohistochemistry staining

The samples were fixed with 4% paraformaldehyde (PFA) containing 2% sucrose in PBS at 4 °C overnight and embedded into paraffin using a tissue processor (EG1150, Leica, Germany). FFPE sections (3 μm thick) were cut with a rotation microtome (RM2255, Leica, Germany). Dewaxed paraffin sections were rehydrated by alcohol series, treated with 3% H_2_O_2_ for 10 min at room temperature and steam-heated for 2.5 min to retrieve the antigen using ethylene diamine tetra acetic acid buffer (PH = 8.0). Subsequent immunostaining was performed with a 50-min incubation period in 37 °C with the monoclonal antibodies for PD-1 and PD-L1 (Supplementary Table S2). The IHC staining of PD-1 and PD-L1 proteins was performed on two different slides. Tonsil tissue was taken as a positive control. Immunoreaction was visualized using a Peroxidase/DAB kit (Cat. K5007, Dako, Denmark). Images were taken with a phase contrast microscope (Eclipse 80i, Nikon, Japan). Details of all reagents with reference to the immunohistochemical staining procedure are listed in Supplementary Table S3.

### Evaluation standard of IHC

The percentages of PD-L1-positive tumour cells and PD-1-positive lymphocytes and staining intensity were evaluated by two pathologists who counted 20 sequential high-power fields (0.54 mm field diameter) judged to be representative of the sample, while remaining blinded to clinical information. Staining intensity was scored as follows: 0 for negative or trace; 1 for weak; 2 for moderate; 3 for high. The percentage of stained cells (0–100%) was multiplied by the dominant intensity pattern of staining ranging from 0 to 3. Therefore, the overall semi-quantitative score (H-score) ranged from 0 to 300 (maximum value of 300 corresponding to 100% of tumour cells positive for PD-L1 or TILs positive for PD-1 with an overall staining intensity score of 3).

### Statistical analysis

The optimal cut-off point for the expression level of each marker including the PD-L1 H-score and GTVnx were determined by the area under the curve (AUC) of the receiver operating characteristic (ROC) curve at the highest positive likelihood ratio point for overall survival (OS). PD-L1 expression was dichotomized into two groups (High and Low), using a cut-off score of ≥155. PD-1 positivity was defined as cases with PD-1 staining intensity ≥2 in more than 5% of TILs.

OS was measured from the date of diagnosis to the date of death or the date of the last follow-up through April 2016. PFS was measured from the date of diagnosis to the date of death or the date of tumour progression (recurrence and/or metastasis) through April 2016. Patients who were still alive at last contact or died from other causes were treated as censored for OS analysis.

A chi-square test was used to assess the correlation of the expression of PD-1 and PD-L1 with various clinical parameters such as age, clinical stage and EBV-DNA status. A t-test was used to compare the differences between the BMI, HB level, and neutrophil and lymphocyte counts in patients with different PD-1/PD-L1 immunoreactivity after a normality test (Kolmogorov-Smirnov test). A non-parametric Mann-Whitney U test was applied to compare the differences in albumin, LDH and the neutrophil/lymphocyte ratio in patients with different PD-1/PD-L1 immunoreactivities. A non-parametric Mann-Whitney U test was also applied to compare the differences in the PD-1/PD-L1 H-score in patients with different baseline EBV-DNA status. The number that follows the ± sign is the standard error (s.e.m.) in this article. Survival analysis was depicted by the Kaplan-Meier method defined by cut-off points generated from the ROC. Univariate analyses and multivariate analyses were performed with a log-rank test and Cox regression analysis, respectively. A P value < 0.05 was used to denote statistical significance, and all reported P values are two sided. P values were marked as *P < 0.05, **P < 0.01, ***P < 0.001, indicating different level of significance. These statistical analyses were performed with SPSS 16.0 48,49,50,51,52,53,54,55,56,57,58,59.

## Additional Information

**How to cite this article**: Zhou, Y. *et al*. PD-L1 predicts poor prognosis for nasopharyngeal carcinoma irrespective of PD-1 and EBV-DNA load. *Sci. Rep.* **7**, 43627; doi: 10.1038/srep43627 (2017).

**Publisher's note:** Springer Nature remains neutral with regard to jurisdictional claims in published maps and institutional affiliations.

## Supplementary Material

Supplementary Information

## Figures and Tables

**Table 1:** Clinico-pathologic variables and immunoreactivity status of PD-1 and PD-L1 in 99 NPC patients.

Variables		n	PD-1 Positive	P value^1^	PD-L1 High^2^	P value^1^
Gender	Male	68	30 (44. 1%)	0.923	44 (64.7%)	0.349
	Female	31	14 (45.2%)		17 (54.8%)	
Age (years)	<50	60	27 (45.0%)	0.890	35 (58.3%)	0.405
	≥50	39	17 (43.6%)		26 (66.7%)	
Smoking status	Smoker or ex-smoker	34	15 (44.1%)	0.962	20 (58.8%)	0.679
	Non-smoker	65	29 (44.6%)		41 (63.1%)	
Family history	Yes	25	14 (56.0%)	0.179	13 (52.0%)	0.253
	No	74	30 (40.5%)		48 (64.9%)	
Tumour size	rT1-3	71	33 (46.5%)	0.517	43 (60.6%)	0.732
	rT4	28	11 (39.3%)		18 (64.3%)	
Nodal status	N0-1	54	22 (40.7%)	0.417	33 (61.1%)	0.910
	N2-3	45	22 (48.9%)		28 (62.2%)	
Clinical stage^3^	I-III	65	29 (44.6%)	0.962	38 (58.5%)	0.372
	IV	34	15 (44.1%)		23 (67.6%)	
GTVnx (mm^3^)^2^	<29.6	45	22 (48.9%)	0.417	28 (62.2%)	0.910
	≥29.6	54	22 (40.7%)		33 (61.1%)	
EBV-DNA load	Undetectable	22	10 (45.5%)	0.914	12 (54.5%)	0.439
	Detectable	77	34 (44.2%)		49 (63.6%)	
PD-1 positivity	Negative	55			31 (56.4%)	0.230
	Positive	44			30 (68.2%)	

^1^Chi-square test.

^2^The optimal cut-off points for PD-L1 and GTVnx were determined by the receiver operating characteristic (ROC) curve for overall survival (OS).

^3^According to the 7^th^ Edition of the AJCC/UICC Staging System for Nasopharyngeal Cancer.

**Table 2:** Univariate analysis of prognostic factors involved in survival.

		Cases (n = 99)	PFS		OS	
Variables			X^2^	P value^1^	X^2^	P value^1^
Gender	Male	68	0.725	0.394	0.344	0.558
	Female	31				
Age (years)	<50	60	2.327	0.127	5.551	0.018*
	≥50	39				
Tumour size	rT1-3	71	5.030	0.025*	6.959	0.008**
	rT4	28				
Nodal status	N0-1	54	2.227	0.136	0.005	0.941
	N2-3	45				
Clinical stage^3^	I-III	65	2.306	0.129	4.372	0.037*
	IV	34				
GTVnx (mm^3^)^2^	<29.6	45	5.049	0.025*	8.090	0.004**
	≥29.6	54				
EBV-DNA load	undetectable	22	0.324	0.569	0.005	0.942
	detectable	77				
Treatment	IMRT	10	2.273	0.132	1.020	0.312
	IMRT+CT	89				
PD-1 positivity	negative	55	0.251	0.616	0.334	0.563
	positive	44				
PD-L1 H-score^2^	<155	38	2.327	0.127	5.870	0.015*
	≥155	61				

^1^Log-rank test.

^2^The optimal cut-off points for GTVnx and the PD-L1 H-score were determined by the receiver operating characteristic (ROC) curve for overall survival (OS).

^3^According to the 7^th^ Edition of the AJCC/UICC Staging System for Nasopharyngeal Cancer. ^*^P < 0.05, ^**^P < 0.01, ^***^P < 0.001.

**Table 3:** Multivariate analysis of prognostic factors involved in survival.

Variables	β	P value^1^	Hazard ratio	95% confidence interval
Age	0.051	0.130	1.053	0.985–1.125
Clinical stage (I-III vs IV)^2^	−0.967	0.307	0.380	0.060–2.430
GTVnx	0.017	0.156	1.017	0.994–1.041
PD-L1 H-score	0.016	0.028*	1.016	1.002–1.031

^1^Cox regression analysis. ^2^According to the 7^th^ Edition of the AJCC/UICC Staging System for Nasopharyngeal Cancer. ^*^ P < 0.05.
