# Supplementary material for: PD-L1 predicts poor prognosis for nasopharyngeal carcinoma irrespective of PD-1 and EBV-DNA load
Source: Sci Rep. 2017 Mar 3;7:43627. doi: 10.1038/srep43627 (PMC5335261; doi:10.1038/srep43627)
Supplement: Supplementary Information [file srep43627-s1.pdf]

The title: PD-L1 predicts poor prognosis for nasopharyngeal carcinoma irrespective of PD-1 and EBV-DNA load

The authors: Yajuan Zhou, Dingbo Shi, Jingjing Miao, Haijun Wu, Jiewei Chen, Xiaoyi Zhou, Desheng Hu, Chong Zhao, Wuguo Deng, Conghua Xie

Supplementary Information 1

Supplementary Figure S1. Potential peripheral blood biomarkers in NPC patients with different PD-1 positivity. (a-f) Statistical analysis suggested no significance (n. s.) between PD-1 positivity and neutrophil counts **(a)**, lymphocyte counts **(b)**, neutrophil / lymphocyte ratio **(c)**, platelet **(d)**, lactate dehydrogenase level **(e)** as well as albumin **(f)** in the 99 enrolled NPC patients. A t-test was used to evaluate the association of PD-1 positivity with lymphocyte counts, neutrophil counts and platelet after normality tests and homogeneity of variance test. A non-parametric Mann-Whitney U test was applied to evaluate the association of PD-1 positivity with neutrophil / lymphocyte ratio, albumin and lactate dehydrogenase level.

Supplementary Figure S2. Potential peripheral blood biomarkers in NPC patients with different level of PD-L1 expression. (a-f) Statistical analysis suggested no significance (n. s.) between PD-L1 level and neutrophil counts **(a)**, lymphocyte counts **(b)**, neutrophil / lymphocyte ratio **(c)**, platelet **(d)**, lactate dehydrogenase level **(e)** as well as albumin **(f)** in the 99 enrolled NPC patients. A

t-test was used to evaluate the association of PD-L1 level with lymphocyte counts, neutrophil counts and platelet after normality tests and homogeneity of variance test. A non-parametric Mann-Whitney U test was applied to evaluate the association of PD-L1 level with neutrophil / lymphocyte ratio, albumin and lactate dehydrogenase level.

Supplementary Figure S1. Potential peripheral blood biomarkers in NPC patients with different PD-1 positivity.

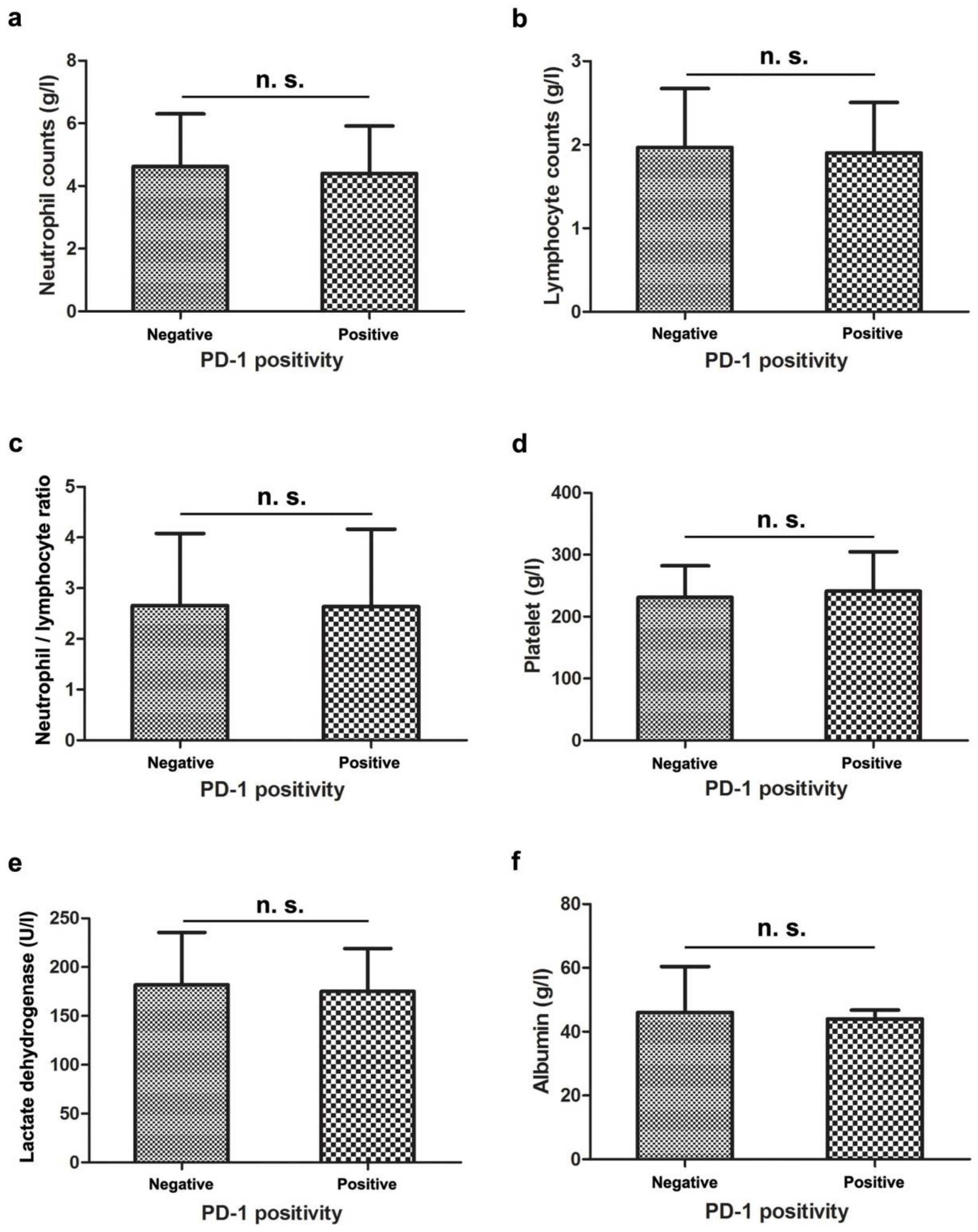

Supplementary Figure S2. Potential peripheral blood biomarkers in NPC patients with different level of PD-L1 expression.

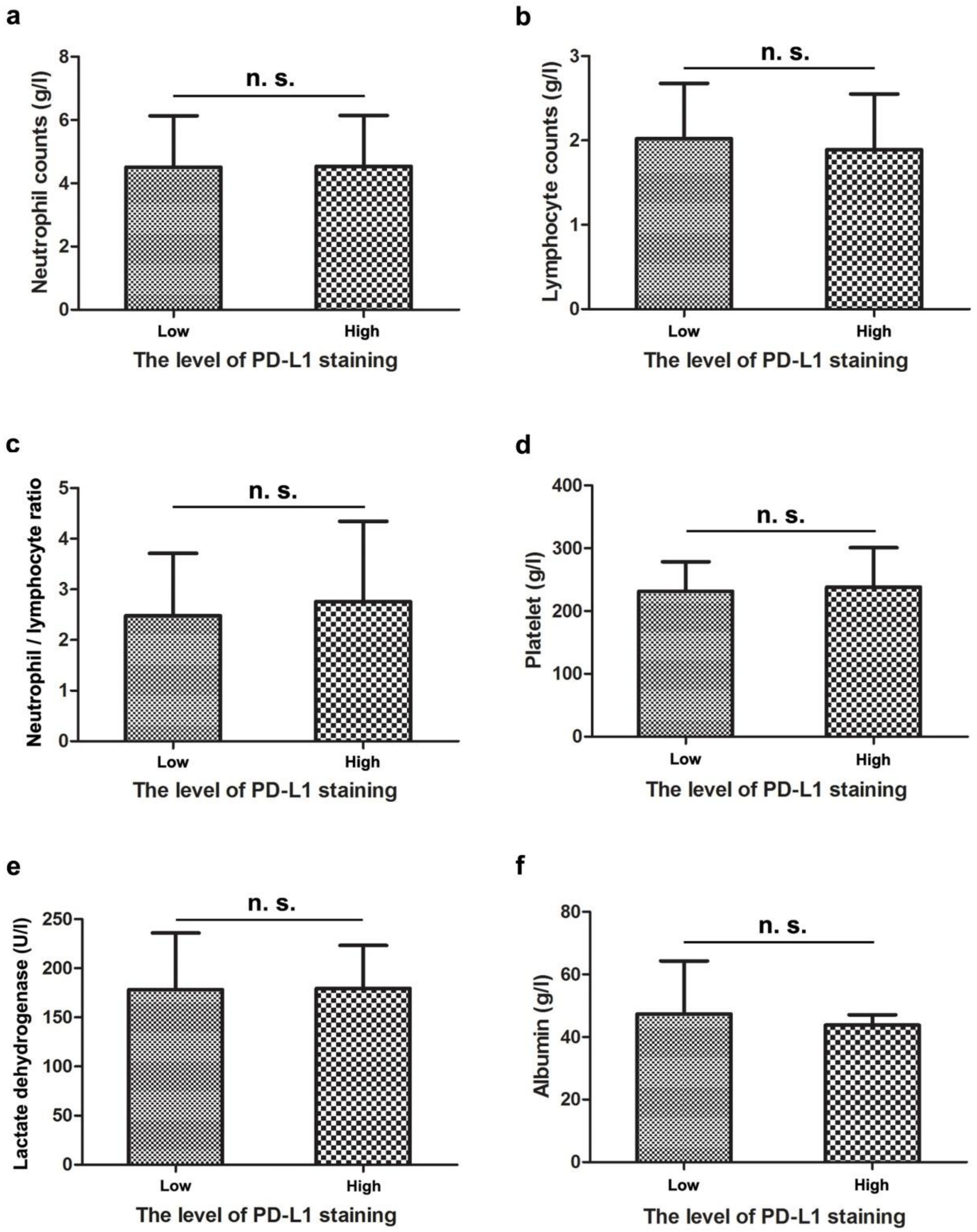

Supplementary Information 2

Supplementary Table S1. Basic clinical characteristics of 99 NPC patients.

[1]According to the 7th Edition of the AJCC/UICC Staging System for Nasopharyngeal Cancer.

Variables		Cases (n = 99)	Percentage (%)
**Follow-up time**	Median (range)	49.4 (6.7-64.9)	
**Age (years)**	Median (range)	47.5 (20-78)	
**Gender**	Male	68	68.7
	Female	31	31.3
**Smoking status**	Smoker or ex-smoker	34	34.3
	Non-smoker	65	65.7
**Family history**	Yes	25	25.3
	No	74	74.7
**Clinical stage[1]**	I	3	3.0
	II	9	9.1
	III	53	53.5
	IV a	23	23.2
	IV b	11	11.1
**Chemotherapy**	No	10	10.1
	Inductive	2	2.0
	Concurrent	28	28.3
	Inductive + concurrent	59	59.6
**Survival rate**	3-year OS	94	94.9
	3-year PFS	83	83.8
**Treatment failure**	Yes	18	18.2
	No	81	81.8

Supplementary Table S2. Primary antibodies used for immunohistochemistry staining.

**Antigens**	**Species antibodies raised in**	**Dilution**	**Supplier**
**PD-1, human**	mouse, monoclonal	1:100	Abcam, UK, Cat. #ab52587
**PD-L1, human**	rabbit, monoclonal	1:100	Cell Signaling Technology, USA, Cat. #13684

Supplementary Table S3. Details of ancillary reagents for immunohistochemistry staining.

Reagent	Supplier	Catalogue Number	Concentration
PFA	Sangon biotech, China	E672002-0500	4 %
sucrose	Sangon biotech, China	CAS 57-50-1	2 %
PBS	Sangon biotech, China	B548117	property
paraffin	Sangon biotech, China	A601888	property
H_2O_2	Sangon biotech, China	C500069-0250	3 %
Ethylene Diamine Tetra acetic Acid buffer	Sangon biotech, China	B540628-0250	property
Antibody Diluent	Sangon biotech, China	D608502-0005	property
mounting medium	Sangon biotech, China	A600157-0100	property
